# Aggregation Periods Influence Step Count Error in Low-Power Wearables

**DOI:** 10.3390/s25226998

**Published:** 2025-11-16

**Authors:** Sydney Lundell, Kenton R. Kaufman

**Affiliations:** Mayo Clinic Motion Analysis Laboratory, Rochester, MN 55905, USA; lundell.sydney@mayo.edu

**Keywords:** wearable sensors, step count accuracy, low power wearables, data aggregation, walking bouts, free living validation, bin size, aggregation periods

## Abstract

**Highlights:**

**What are the main findings?**

- Aggregating step count into large aggregation periods significantly increases error and underestimation of step count in lower-power wearables
- Aggregation periods containing continuous walking bouts spanning the entire period are more accurate than aggregation periods containing multiple non-continuous walking bouts.

**What are the implications of the main findings?**

- Selection of the time aggregation period is critical for accurate step detection in a free-living environment, particularly for clinical and compliance monitoring.
- Optimizing aggregation periods for the metric of interest can improve wearable sensor performance without compromising battery life.

**Abstract:**

Wearable sensors are increasingly used to monitor physical activity, yet low-power devices often rely on data aggregation to conserve battery life, potentially impacting measurement accuracy. This study evaluates the performance of a new low-power wearable (LPW), designed for monitoring steps across multiple months in a free-living environment, compared to a research-grade sensor (RGS) that collects raw acceleration data, with a focus on how different aggregation intervals impact step count accuracy. Thirty-two participants wore both sensors over two days, with LPW data collected in 10 min, 1 min, or 10 s aggregation periods (APs). Sensitivity and specificity of wear time detection were high across all APs (0.96 and 0.98, respectively). While total daily step count error did not differ significantly between APs, the 10 min AP exhibited greater undercounting and wider limits of agreement, especially in APs containing more than 40 steps. These findings suggest that although AP does not affect total daily step count, it influences the accuracy and variability of more granular data windows. Aggregating step counts over longer intervals may obscure short, fragmented bouts common in daily activity, leading to underestimation of steps. Optimizing APs and sensor settings is critical for improving accuracy in low-power wearables used outside laboratory settings.

## 1. Introduction

Wearable devices are growing in popularity for (a) monitoring individual physical activity and (b) managing the health needs of a population that continues to become older and outnumber practicing physicians [1,2,3,4]. Movement and activity—which are known to be directly correlated to overall physical, cardiovascular, and mental health—are the primary focus of these wearable health monitors [5]. The advantage of these sensors is in their ability to track changes in activity more accurately than patient journals and increase user activity when personalized summaries are provided [6].

Low power wearable sensors must balance competing factors such as battery life and frequency of data collection [7]. These sensors mitigate the challenge of battery life by processing data using analog signal processing embedded in the sensor along with predetermined digital settings to calculate metrics such as steps/min or heart rate instead of recording the raw signal at a high frequency. As a result, abnormal metrics such as slower-than-average steps (e.g., <100 steps/min), or short step bouts (e.g., <4 steps), are commonly filtered from the raw signal, resulting in a total step count that is lower than the true total [8]. This is particularly important, as underestimation of steps has been shown to have a negative impact on user activity and perceived health [9]. In order to mitigate the likelihood of undercounting, Lundell et al. have developed a method for optimizing low power sensor settings for populations that present with slower walking speeds [10]. This method minimizes miscounted steps by optimizing sensor settings for the range of walking speeds seen in a population. However, this method has only been conducted in a laboratory setting. Validation of sensor accuracy during activities of daily living (ADL) is often performed in controlled environments to minimize external noise, yet this approach fails to account for the influence of aggregation periods, routinely employed in clinical sensors, on measurement accuracy [9,11,12]. Further validation is needed in a free-living environment to determine the sensor’s true accuracy outside of a laboratory, where environmental noise is known to decrease wearable sensor accuracy [13]. Currently the accuracy of different data acquisition APs has not been assessed in low-power wearable step-counting devices outside of a controlled environment.

This study aims to evaluate the performance and accuracy of a novel low power wearable sensor (LPW) outside of a controlled laboratory environment compared to a research grade sensor (RGS) while examining how AP impacts accuracy. First, the sensitivity and specificity of the sensor’s wear time metric will be assessed to determine its reliability in detecting wear time as a compliance metric. Next, the study will investigate whether the aggregation of data—specifically, 10 min vs. 1 min vs. 10 s intervals—affects the LPW sensor’s ability to accurately capture total daily step count as well as the accuracy of step count per bin. Finally, the impact of short, non-continuous bouts of walking compared to continuous bouts spanning multiple APs affects step-count accuracy. We anticipate that this comprehensive evaluation will establish the validity of a LPW for measuring physical activity in an able-bodied population and outline the potential impact aggregation periods can have on low power wearable accuracy.

## 2. Materials and Methods

### 2.1. Data Collection

A total of 32 participants (20 female) aged 28 ± 6 years old were recruited by word of mouth and institutional advertisement. Participants were consented prior to data collection in person or over email by study staff. Data from two subjects were excluded from analysis due to sensor failure and three were removed due to failure to complete their collection.

**Figure 1:**
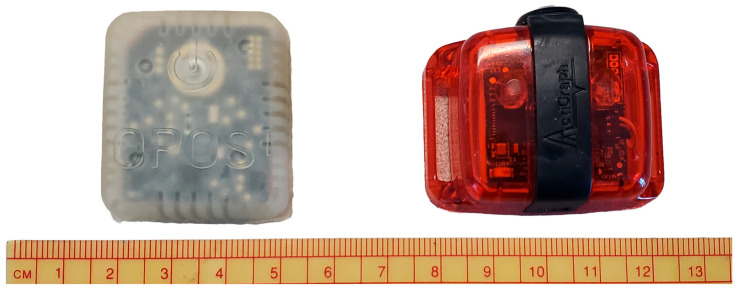
OPOS sensor (**left**) and ActiGraph sensor (**right**) pictured above a ruler for size.

Two sensors were used in the study (Figure 1). The research grade sensor (RGS), Actigraph wGT3X-BT sensor (Ametris, Pensacola, FL, USA) and the medical grade sensor (LPW), OPOS1 (OPOS1, Waterloo, IA, USA). Each participant wore a total of 5 sensors: one RGS on their waist and two sensors, one RGS and one LPW, on each ankle. The RGS were affixed to the participant via adjustable Velcro straps while the LPW sensor was tucked into the sock above the RGS. Participants were instructed to don the sensors at the start of their day and remove them for activities they would not wear their shoes for (e.g., Swimming, showering) and when they went to bed.

The LPW operates in a low-power inertial measurement unit (IMU) mode to estimate total step count from accelerations collected at 26 Hz. This mode relies on three key parameters: threshold, debounce time, and debounce steps which were previously optimized by Lundell et al. [10]. To minimize power draw (caused by writing data to a single datapoint) and increase the sensor’s lifetime, steps are aggregated into bins. For this project, three versions of the software compiled data into 10 min, 1 min, or 10 s APs. Fifteen subjects received sensors that collected data over a 10 min sampling period, six subjects collected data at a 1 min AP, and the remaining nine subjects collected data at a 10 s AP. The uneven distribution of these groups was due to the staggered release of software corresponding to subject recruitment, but an appropriate sample size calculated by the methods in Lu et al. [14] was collected for each AP.

The RGS collected raw accelerations at 50 Hz over 48 h. This raw data was exported to MATLAB (2020b, MATLAB, The MathWorks Inc. Natick, MA, USA) where it was processed using methods developed by Fortune and Lugade et al. to calculate step count, activity, levels, and cadence [15]. These metrics were then compiled into groups of 10 min, 1 min, or 10 s APs corresponding to the LPW sensor AP.

### 2.2. Data Processing

#### 2.2.1. Wear Time

The sensitivity and specificity for each subject was calculated and grouped into an AP. Normality was tested for both groups using a quartile–quartile plot. A Kruskal–Wallis test was used to test for significant differences in sensitivity/specificity between the AP sizes as the sample size was small, not equal, and not normally distributed [16]. The median and IQR for the combined dataset of all three APs was then calculated.

#### 2.2.2. Total Step Count per Day

Total step count per day was calculated for each subject using the RGS as the ground truth. The resulting percentage error from the LPW and RGS was then calculated. A one-way ANOVA was conducted across the three aggregation frequencies to determine if there was a significant difference between errors in total step count and the APs. Pearson’s correlation coefficient and *p*-values were calculated for the percent error and magnitude of steps.

#### 2.2.3. Aggregation Period Accuracy

Bland–Altman plots were used to assess the bias and limits of agreement between the LPW and RGS. A combined Bland–Altman plot was generated to better visualize the overlap for each TAP sample. Although the Bland–Alman plot is a combination of all the bins, the bias and limits of agreement were calculated individually and rounded to the nearest step. For each Bland–Altman plot, a supplementary bar chart was included to represent the magnitude of repeated measurements [17]. The limits of agreement were plotted on the bar chart to better visualize what percentage of all datapoints fell within the bounds. Because Bland–Altman plots are largely visual and depend on predefined limits, Lin’s concordance correlation coefficient (LCCC) (95% CI) was also calculated to quantify agreement. To address heteroscedasticity in total steps, a piecewise Bland–Altman analysis was performed using step-frequency ranges defined by Orendurff [18] across all bins, AP pairs, and individual APs to assess if any AP size significantly altered the limits of agreement.

#### 2.2.4. Continuous and Non-Continuous Walking Bouts

Walking segments were identified using continuous wavelet transform, following Fortune and Lugade et al. [15]. APs that contained a single walking segment spanning the entire period were considered continuous. APs where a single walking segment occurred but did not span its entirety was included if the portion of the segment within the AP was more than 4 steps—the LPW threshold for identifying steps. A Bland–Altman plot with bias and limits of agreement was calculated for each group, as was the corresponding LCCC.

## 3. Results

### 3.1. Wear Time

**Figure 2:**
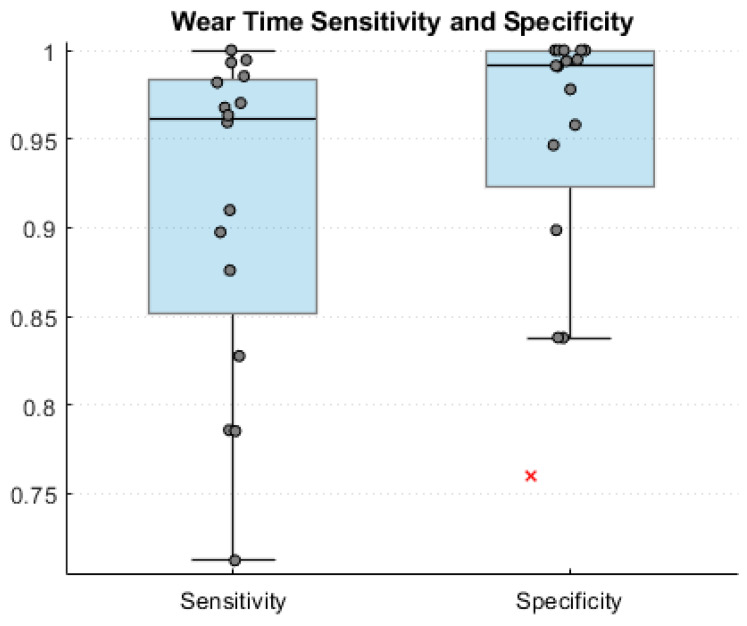
Wear-time sensitivity and specificity for all participants. A single outlier (0.76) in specificity is marked by a red x.

There was no significant difference across AP size for sensitivity (*p* = 0.57) and specificity (*p* = 0.84). Accordingly, the sensitivity and specificity metrics for all wear times were compiled into a single calculation of sensitivity and specificity. The median and IQR of the sensitivity and specificity of the LPW in relation to the RGS’s calculated wear time were 0.96 (0.85, 0.98) and 0.98 (0.92, 1), respectively (Figure 2).

### 3.2. Total Steps per Day

**Figure 3:**
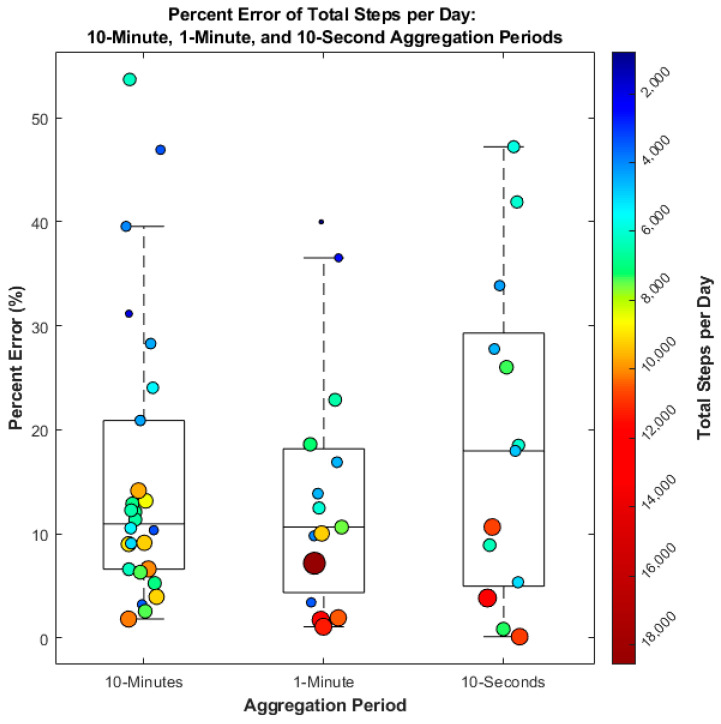
Percent error in total daily step counts across three data aggregation intervals: 10 min, 1 min, and 10 s aggregation periods. Each point represents a day of data collection, with marker size and color corresponding to total daily steps (as indicated by the color bar). Larger, red markers represent higher total step counts, while smaller, blue markers indicate lower step counts. data points are fewer total steps.

A variable sample size was present across AP sizes over the two-day period: 909 samples for the 10 min AP, 3356 samples for the 1 min AP, and 4813 samples for the 10 s AP. A total of 3391 data points with zero steps were excluded from the 10 s analysis as these APs were during times where movement was detected but not steps. There was no significant difference (*p* = 0.94) in the errors in total step counts across the 10 min, 1 min, or 10 s AP. Error was inversely correlated (r = −0.73), (*p* = 0.014) to the magnitude of total steps, suggesting a higher accuracy with more steps taken. The median and IQR of percent error for the 10 min AP were 11 (7, 21)%, 11 (4, 18)% for 1 min, and 18 (5, 30)% for 10 s (Figure 3).

### 3.3. Aggregation Period Accuracy

**Figure 4:**
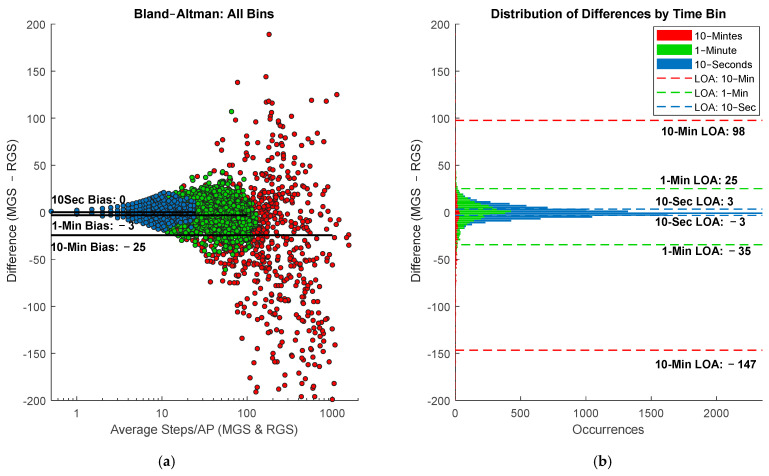
Bland–Altman plot comparing step counts between RGS and LPWs across three AP sizes: 10 min, 1 min, and 10 s. (**a**) Superimposed on the combined scatter plot are the biases calculated for each AP which are 0, −3.2, and −24.5 steps for the 10 s, 1 min, and 10 min AP, respectively. The corresponding histograph (**b**) depicts the number of occurrences for each AP to highlight the limited resolution of the Bland–Altman plot, particularly in APs of 10 s and 1 min. Also shown on the histograph are the limits of agreement for each AP with the 10 s AP generating the smallest LOA from −3.4 steps to 3.3 steps.

A total of 3391 data points with zero steps were excluded from the 10 s analysis as these APs were during times where movement was detected but not steps. As AP size increased, the bias in step count decreased. The 10 min Bland–Altman plot revealed a tendency of the LPW to undercount by an average of −25 steps (Figure 4a) with wide limits of agreement ranging from −147 to 98 steps (Figure 4b). Notably, the 10 min AP also yielded the highest LCCC of 0.96 (95% CI: 0.956 to 0.965). In comparison, smaller AP sizes showed reduced bias: the 1 min AP had a bias of −3 steps and limits of agreement ranging from −35 and 25 steps, while the 10 s AP showed no bias and limits of agreement ranging from −3 to 3 steps. Corresponding LCCC values were 0.91 (95% CI: 0.90 to 0.92) for the 1 min AP and 0.79 (95% CI: 0.79to 0.8) for the 10 s AP.

The bias and limits of agreement (LOA) between step counts recorded by different AP sizes (10 s, 1 min, and 10 min) were calculated across ranges of total steps per AP (Table 1). In lower step count ranges (0–10 and 10–20), biases were generally small for all AP sizes, although the 10 s AP consistently underestimated steps (e.g., −2 in 0–10 bin). Agreement began to diverge more substantially with increasing step counts. In the 20–40 range, the 10 min AP exhibited a noticeably higher negative bias (−8) and wider LOA compared to shorter AP sizes. This pattern became more pronounced at higher step counts. For example, in the 40–100 step/AP range, the 10 min AP showed a large bias of −13 and a wide LOA (−81 to 45), significantly higher than shorter bins. At even higher step/APs (100–200 and beyond), data collected exclusively with 10 min APs reveals a consistent negative bias (e.g., −15 in 100–200, −30 in 200–400), suggesting substantial underestimation. Notably, biases involving the 10 min AP are consistently more negative, and in APs exceeding 200 steps, LOA becomes extremely wide (e.g., ±100+), indicating poor agreement. Cells marked with † indicate limited data from single-AP configurations.

### 3.4. Continuous and Noncontinuous Walking Bouts

**Figure 5:**
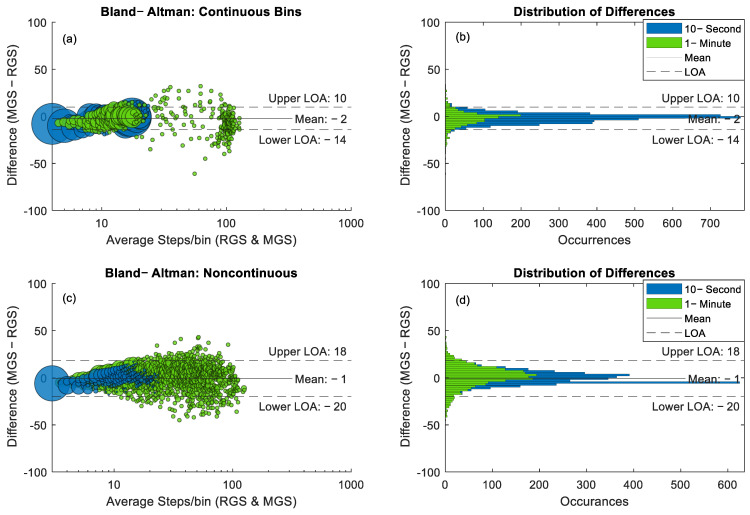
Bland–Altman plots comparing the difference in step counts between the RGS and LPWs where data points were separated by continuous walking bouts that were contained in a single AP (**a**) and noncontinuous walking bouts within an AP (**c**). Datapoint size within the Bland–Altman plots corresponds to the number of occurrences at that datapoint. The corresponding histograms to the right of each plot (**b**,**d**) provide additional context for the density and spread of walking bouts and their corresponding AP size.

Comparisons were made of APs containing a single steady-state walking bout (Figure 5). No continuous walking bouts exceeded 10 min in length; therefore, none were included in the analysis comparing continuous and noncontinuous bins. Continuous walking bouts exhibited a negligible undercounting bias of −2 steps, with limits of agreement (LOA) ranging from −14 to 10 steps (Figure 5a,b). Within the continuous bins, only 70 instances (1.5%) fell within the 25–80 step range, compared to 27% of noncontinuous bouts (Figure 5c,d). Noncontinuous bouts also showed a negligible bias of −1 step, with a LOA spanning from −20 to 18 steps representing a 59% increase in the LOA span compared to continuous bouts. LCCC for the noncontinuous bouts was 0.91 with a 95% confidence interval of 0.911, 0.922. For continuous bouts LCCC was 0.95 (0.950, 0.955).

## 4. Discussion

This study demonstrated that error can be exacerbated by different APs in low power wearable sensors outside of a controlled laboratory environment. The LPW showed no significant change in sensitivity 0.96 (0.85,0.98) (*p* = 0.57) or specificity 0.98 (0.92,1) (*p* = 0.84) across AP size. Similarly, there was no significant difference in median error of total steps per day in any AP sizes, and the LPW undercounted steps, on average, by 13% during ADL, which agrees with the current literature [11,19,20]. Intuitively there should be no significant change caused by AP, as aggregation is simply the summing of a metric generated from a raw signal over a given period, but this study has shown that aggregation periods can either magnify or minimize specific error sources embedded in the filtering process. Factors such as walking speed and wear location have been found to significantly impact the accuracy of wearable sensors with ankle and thigh generally providing more accurate measurements [9,21]. Low power wearable sensors specifically, which utilize onboard processing to calculate a desired metric, struggle to accurately collect steps during slow walking speeds in a free-living environment [20,21]. This study found that, in a healthy population, walking bout continuity spanning the duration of an AP is also a factor in low power wearable sensor accuracy.

Aggregating step counts into 10 min APs or larger is a technique used to manage storage limitations and maximize battery life. To address battery life and storage limitations, the LPW aggregates step counts into 10 min APs to maximize data collection over a long period of time. This trade-off results in the 10 min intervals exhibiting the widest range of possible step values with the distribution of values following a lognormal distribution that is characteristic of normal bouts [18]. Alternative configurations using 1 min and 10 s APs prioritize finer temporal resolution, enabling analysis of individual bouts, while sacrificing battery life and decreasing the range of possible step values.

Despite these different priorities, no significant difference in total step count error across different AP sizes suggesting that aggregation frequency alone does not impact accuracy of total steps taken in a day (Figure 3). However, a strong negative correlation was observed between the total number of steps taken per day and step count error (r = −0.73), indicating that individuals with higher daily step counts experienced lower measurement errors. This trend may be attributed to the onboard step detection algorithm, which relies on preset debounce parameters. Specifically, the debounce time (the maximum time interval allowed between steps) and debounce step minimum (the threshold number of steps required to classify a bout) are designed to filter out non-purposeful movements such as fidgeting or shuffling [10]. While effective for excluding noise, these settings may also inadvertently discard valid steps, particularly those occurring at lower speeds, which are more common in older populations, and during turning or on hazardous surfaces [22,23].

Orendurff et al. has shown that people do not walk in long continuous groups of steps but instead short bursts that 40% of the time last no more than 20 s, suggesting a 10 min period, could have up to 30 bouts of 20 s in length where steps are erroneously excluded [18]. The inability to distinguish between 10 min periods consisting of a few long bouts versus numerous short bouts further complicates interpretation. This limitation is reflected in the accuracy analysis across AP sizes. While average total step count per day error did not differ significantly between AP sizes, the 10 min APs exhibited substantially wider limits of agreement and increasingly negative bias as total steps per AP increased. For instance, at step ranges of 40–100 and above, the 10 min AP showed much larger underestimations (a bias of −13 steps in bouts containing 40–100 steps) and greater variability than the 1 min and 10 s APs. This reduced agreement likely results from the higher frequency of short, noncontinuous bouts aggregated within each 10 min window.

Accuracy of step counting in traditional wearable sensors is highest when steps are of a consistent frequency, such as during running, or are continuous over multiple minutes [11,16]. Unfortunately, humans do not move in long continuous walking bouts and instead take 40% of their steps over small segments of less than 12 ± 1 steps [15]. Wearable sensor error increases with ADL due to the inconsistency in walking segments during these tasks [11] as well as decreased walking speeds [21,24]. As anticipated, only 1 min and 10 s datapoints were identified to contain APs of continuous bouts. Bias was −2 steps for continuous and −1 step for noncontinuous bouts, with noncontinuous bouts showing a 150% wider LOA range. This increase in variability is echoed in the bin-level comparison, where 10 min APs consistently showed greater undercounting and wider LOA compared to the 1 min and 10 s intervals, particularly in APs containing more than 40 steps (75% of walking bouts are less than this) [18]. This likely results from short, fragmented bouts within 10 min windows, which are more prone to undercounting due to LPW debounce settings. In contrast, 1 min APs better preserve walking continuity while balancing battery efficiency in low-power devices.

This validation study has some limitations. The participants sampled were primarily young adults with sedentary jobs. This limitation was mitigated by collecting over two days, one weekday and one weekend, to capture a broader range of activities. Additionally, synchronization between systems was challenging, particularly during 10 s, where manual sync errors appeared as consistent step undercounts that shifted to the final bin. Additionally, the LPW’s real-time clock is susceptible to a 0–7 s drift per day. Realignment of the RTC of the LPW occurs each time data is downloaded from the sensors, but over long periods of time can cause misalignment to present in 10 s APs in particular, but has an insignificant impact on step count for large APs of 1–10 min in size.

## 5. Conclusions

This study highlights the importance of considering AP size in low power wearable devices, not just for managing battery life, but in mitigating the influence of embedded settings on step count accuracy. Consistent undercounting bias, particularly during short bouts, can occur with existing sensors on the market. Importantly, a sensor’s settings contribute to undercounting as well as the duration of data aggregation. Adjusting these parameters should improve accuracy in individual bins. Future research should explore sensor performance in diverse populations and assess ability to detect step count in disabled populations. Despite its limitations, the LPW presents a reliable option for step tracking and compliance monitoring, supporting its potential utility in both clinical and everyday settings.

## Figures and Tables

**Table 1:** Comparison of Bias and LOA across different total steps/AP and the AP sizes. The calculated biases and limits of agreement (LOA) for the total dataset along the 6 ranges (0–10, 10–20, 20–40, 40–100, 100–200, 200–400) is recorded along the second column. From 0 to 200 steps the biases and LOA are calculated from a combination of all three AP sizes. Individual cells labeled with a † contain only datapoints collected within a single AP making analysis of AP size on bias and LOA impossible at a scale where more than 200 steps were recorded in a bin. The same is true for 1 min APs within cells that are labeled with † making direct comparison to unnecessary cells. Within the 10 min AP for step counts between 40 and 100 (*) the limits of agreement are 70.65% larger than the LOA calculated with all three AP sizes. The bias is also 9 steps lower in the 10 min AP.

Range of Total Steps/Bin	All Bins Bias (LOA)	10-Min Bias (LOA)	1-Min & 10-Min Bias (LOA)	1-Min Bias (LOA)	10-s & 1-Min Bias (LOA)	10-s Bias (LOA)	10-Min & 10-s Bias (LOA)
0–10	0 (−10, 9)	−1 (−9, 7)	0 ^†^ (−12, 11)	0 * (−12, 11)	−2 (−11, 8)	−2 (−11, 8)	−2 (−11, 8)
10–20	0 (−11, 11)	−1 (−25, 24)	1 (−14, 16)	1 (−14, 15)	−1 (−20, 11)	−1 (−10, 8)	−1 (−10, 8)
20–40	1 (−24, 27)	−8 (−43.0, 27)	1 (−26, 28)	3 (−22, 27)	3 (−20, 25)	3 (−7, 11)	−3 (−31, 25)
40–100	−4 (−49, 40)	−13 * (−81, 45)	−4 (−49, 40)	−1 (−33, 30)	−1 ^†^ (−33, 30)	NA	−13 ^†^ (−81, 54)
100–200	−15 (−95, 66)	−19.0 (−123, 85)	−14.8 (−95.5, 66)	−9.0 (−36, 8)	−9.0 ^†^ (−36, 8)	NA	−19 ^†^ (−123, 85)
200–400	−30 ^†^ (−162, 101)	−30 (−162,101)	−30 ^†^ (−162, 101)	NA	NA	NA	−30 ^†^ (−162, 101)

## Data Availability

Due to confidentiality agreements, supporting data can only be made available to bona fide researchers subject to a non-disclosure agreement.

## Abbreviations

The following abbreviations are used in this manuscript:

AP	Aggregation Period
ADL	Activities of Daily Living
RGS	Research Grade Sensor
IMU	Inertial Measurement Unit
LCCC	Lin’s Concordance Correlation Coefficient
LOA	Limits of Agreement
